# Machine Learning Driven Contouring of High-Frequency Four-Dimensional Cardiac Ultrasound Data

**DOI:** 10.3390/app11041690

**Published:** 2021-02-13

**Authors:** Frederick W. Damen, David T. Newton, Guang Lin, Craig J. Goergen

**Affiliations:** 1 Weldon School of Biomedical Engineering, Purdue University, West Lafayette, IN 47907, USA; 2 Department of Statistics, Purdue University, West Lafayette, IN 47907, USA; 3 Department of Mathematics & School of Mechanical Engineering, Purdue University, West Lafayette, IN 47907, USA

**Keywords:** echocardiography, 4D ultrasound, volumetric imaging, murine, left ventricle, myocardium, cardiac kinematics, hypertrophic cardiomyopathy, boundary prediction, machine learning

## Abstract

Automatic boundary detection of 4D ultrasound (4DUS) cardiac data is a promising yet challenging application at the intersection of machine learning and medicine. Using recently developed murine 4DUS cardiac imaging data, we demonstrate here a set of three machine learning models that predict left ventricular wall kinematics along both the endo- and epi-cardial boundaries. Each model is fundamentally built on three key features: (1) the projection of raw US data to a lower dimensional subspace, (2) a smoothing spline basis across time, and (3) a strategic parameterization of the left ventricular boundaries. Model 1 is constructed such that boundary predictions are based on individual short-axis images, regardless of their relative position in the ventricle. Model 2 simultaneously incorporates parallel short-axis image data into their predictions. Model 3 builds on the multi-slice approach of model 2, but assists predictions with a single ground-truth position at end-diastole. To assess the performance of each model, Monte Carlo cross validation was used to assess the performance of each model on unseen data. For predicting the radial distance of the endocardium, models 1, 2, and 3 yielded average R^2^ values of 0.41, 0.49, and 0.71, respectively. Monte Carlo simulations of the endocardial wall showed significantly closer predictions when using model 2 versus model 1 at a rate of 48.67%, and using model 3 versus model 2 at a rate of 83.50%. These finding suggest that a machine learning approach where multi-slice data are simultaneously used as input and predictions are aided by a single user input yields the most robust performance. Subsequently, we explore the how metrics of cardiac kinematics compare between ground-truth contours and predicted boundaries. We observed negligible deviations from ground-truth when using predicted boundaries alone, except in the case of early diastolic strain rate, providing confidence for the use of such machine learning models for rapid and reliable assessments of murine cardiac function. To our knowledge, this is the first application of machine learning to murine left ventricular 4DUS data. Future work will be needed to strengthen both model performance and applicability to different cardiac disease models.

## 1. Introduction

As heart disease remains the number one cause of death in the United States [1], echocardiography remains an integral tool to the proper diagnosis and prognosis of abnormal cardiac function. Furthermore, the development of murine models of cardiac disease have provided researchers a strong foundation to further our understanding of pathological hallmarks and how specific genetic and/or environmental factors might drive progression [2-6]. To bridge the gap between imaging technology and murine disease models, high-frequency ultrasound uses MHz frequency ultrasonic waves to acquire images of small structures (e.g., mouse left ventricle with a thickness of ~1 mm) that are rapidly moving (e.g., mouse heart rate is ~500–600 bpm), thus too difficult to be adequately viewed using clinical ultrasound systems. Recent advancements in high-frequency ultrasound technologies have also introduced a collection of four-dimensional ultrasound (4DUS) approaches, allowing for more thorough analyses of cardiac motion beyond global metrics based on idealized geometries (i.e., ejection fraction, stroke volume), similar to that commonly reported with clinical cine-magnetic resonance imaging (cine-MRI) [7-9].

In tandem with the advancement of ultrasound imaging technology, integrations of machine learning and artificial intelligence algorithms—particularly deep neural nets—have shown promise in rapidly and robustly characterizing cardiac kinematics and ultrasound data in general [10-17]. While machine learning has demonstrated notable successes in ventricle segmentation on 4D cardiac MRI data [18-20], epicardial fat segmentation in Computed Tomography (CT) data [21,22], and even boundary detection in clinical 2DUS echocardiography data [10], applications to murine 4DUS data remain limited [12]. This is in part due to unique challenges presented by 4DUS data. Most notably, cardiac US images in mice show lower image contrast in combination with large amounts of speckle noise. Furthermore, as the probe typically covers half of the entire ventral thorax, these artifacts can even renderer manual segmentation difficult. Finally, 4DUS data in general suffer from high dimensionality; even 3DUS segmentation is considered a difficult problem given current tools [10]. The high dimensionality in combination with the relatively small sample sizes commonly seen in medical applications, of which our dataset is no exception, presents even further challenges.

Here, we develop a machine learning model to contour 4DUS data acquired from healthy and diseased (i.e., hypertrophic cardiomyopathy) mice. To our knowledge, this is the first publication of machine learning applied to murine cardiac 4DUS data. While our model is described in detail below, it contains three fundamental components: (1) a low dimensional representation of the raw US data, (2) a basis expansion of time to incorporate the regularity in epicardial movement throughout the cardiac cycle, and (3) the requirement that the model need only predict output values at preselected anchor points using interpolating cubic splines to form the final output structure.

The following section “Materials and Methods” outlines the procedures followed to acquire the data used in training our models, details each model’s composition, and describes how we test each model’s predictive accuracy. The “Results and Discussion” section then demonstrates our quantitative model performance tests’ results and provides context regarding broader applicability and limitations. Finally, we discuss other modelling approaches for future work and potential clinical translatability.

## 2. Materials and Methods

### 2.1. Ultrasound Data

Imaging was performed using a Vevo3100 high-frequency ultrasound system (FUJIFILM VisualSonics Inc., Toronto, ON, Canada) with a 40 MHz center frequency transducer (MX550D) and a translating linear step motor. In preparation for imaging, mice were anesthetized using approximately 1.5–2.0% isoflurane, secured supine on a heated stage with gold-plated electrodes that collected cardiac and respiratory signals, and had hair removed from the ventral surface via a depilatory cream. Each 4DUS dataset was acquired by translating through short-axis slices from below the apex of the heart to above aortic arch, with a sampling frame rate of approximately 300 fps and total scan time of 6–10 min.

A total of 136 4DUS datasets were used to implement the machine learning algorithm, taken from previous studies on genetically-induced cardiac hypertrophy. One effort focused on a mutation of Nkx2-5^183P/+^ [23-25], consisting of 24 mutant and 24 littermate-control 4DUS datasets. The second focused on a mutation of CPT2^M−/−^ [26,27], consisting of 41 mutant and 47 littermate-control 4DUS datasets. While each of these studies does include repeated imaging on some mice at numerous time-points, for the scope of this work each 4DUS scan was treated as independent data. All animal experiments were approved by the Purdue University Institutional Animal Care and Use Committee (protocol code 121100077326; approved 11 December 2015).

### 2.2. 4DUS Analysis and Contour Structure

**Figure 1.**
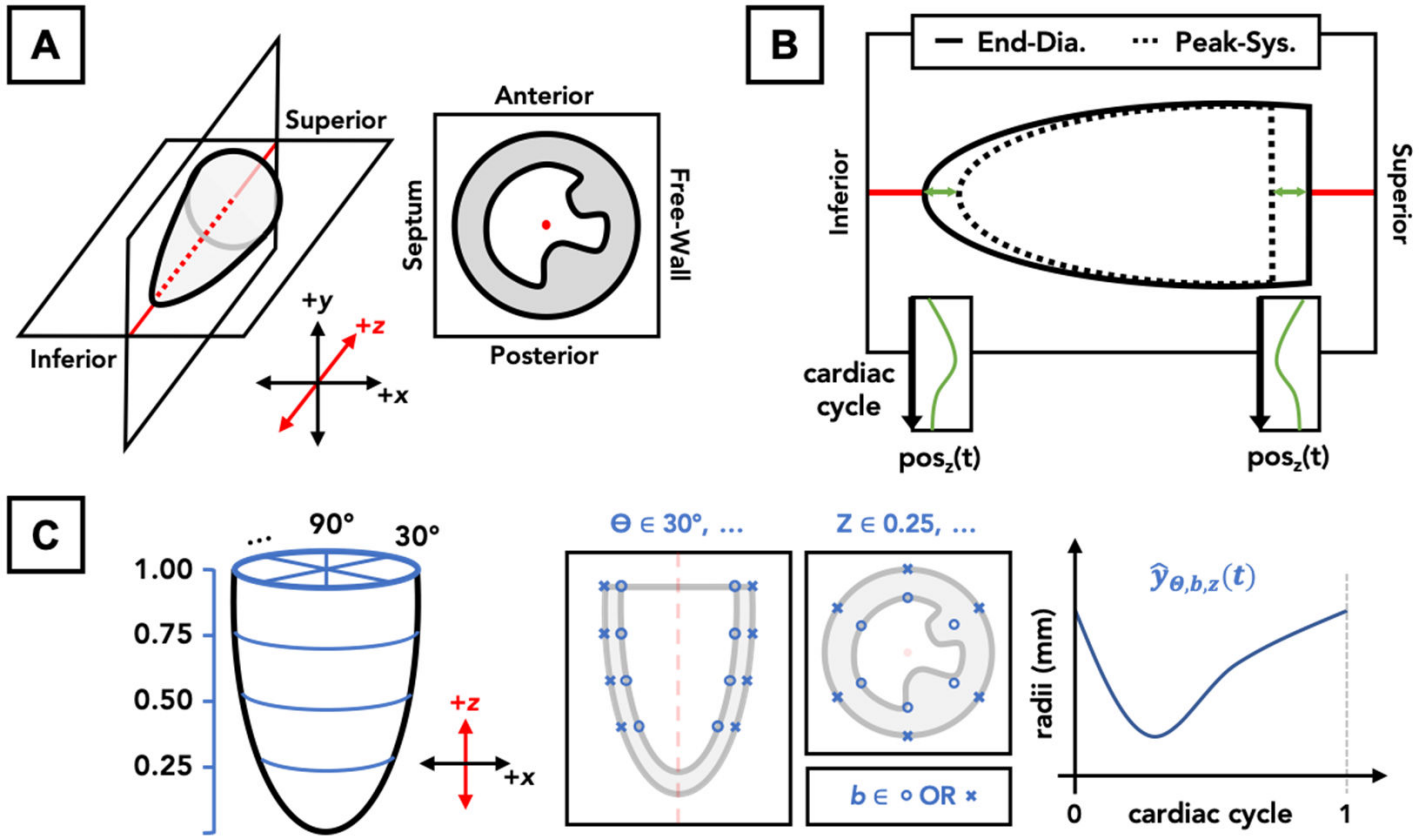
Schematic for post-acquisition analysis on left-ventricular 4DUS data, including: (**A**) spatial reorientation to align with a central *z*-axis (i.e., inferior (−z) to superior (+z)), (**B**) tracking of apex and base locations, and (**C**) definition of boundary points on the endo- and epi-cardial boundaries (Equation (1)), excluding papillary muscles, to be tracked across the cardiac cycle.

Each 4DUS dataset is loaded into a custom interactive toolbox developed in MATLAB (MathWorks Inc., Natick, MA, USA), where data are first reoriented to align to a standard axis and then the endo- and epi-cardial boundaries are manually tracked across a representative cardiac cycle (Figure 1A). The standard axes follow a cartesian coordinate system, and are defined by: (1) the left-ventricular apex and center of the base both fall on the *z*-axis, (2) the anterior and posterior walls fall along the *y*-axis, and (3) the septal wall falls on the negative *x*-axis (i.e., standard radiological orientation). Following reorientation, the *z*-axis location of the apex and base are tracked across the cardiac cycle (Figure 1B). Then, iteratively at each point in time, four equally spaced parallel short-axis slices are interpolated from the reoriented 4DUS data, corresponding to 25, 50, 75, and 100% of the distance from the apex to base. The initial tracking of base and apex locations allows for through-plane motion to be compensated for during subsequent wall-tracking.

In order to create a final three-dimensional mesh of the endo- and epi-cardial boundaries of the left ventricle, a structured subset of points was defined (Figure 1C) such that (1) each of the four parallel slices contains six points for each of the two boundaries, (2) those points are constrained to equally spaced rotations around the central *z*-axis (i.e., 30, 90, 150, 210, 270, and 330 degrees relative to the positive *x*-axis), and (3) the distance between each point and the central *z*-axis is a function of relative time across the cardiac cycle. Once all points are individually repositioned across the cardiac cycle to define the regional kinematics, hobby splines were used to interpolate a three-dimensional mesh of the left-ventricle at a standardized array of cycle-positions. Specifically for this work, the final 4D mesh of the left-ventricle included 60 locations around the *z*-axis, 60 locations from the apex-to-base along the *z*-axis, and 60 time-points across the cardiac cycle.

### 2.3. Machine Learning Algorithms

#### 2.3.1. Prediction Objective

In this context, a machine learning model takes a given 4DUS image as input and returns two predicted 3D surfaces (one for the endocardial boundary, one for the epicardial boundary) for each timepoint. The nature of the task immediately presents several difficulties: the data are high dimensional (160,000 pixels per image with about 5000 total images), US data naturally contain speckle noise, and the model output consists of two smooth 3D surfaces across time with no specified parametric form. The data processing approach described in Section 2.2 significantly reduces the problem complexity while still allowing for flexible contours to be estimated. Predicting radial distances of each anchor point becomes a regression problem, with the complete contours being inferred using smoothing splines after the anchor points have been estimated.

#### 2.3.2. Modeling Approach

In order to manage the high dimensionality of the image data, principal component analysis (PCA) was used to project the US images onto a lower dimensional subspace. Since the selected anchor points have fairly regular movement patterns, we further incorporated smoothing splines to capture the average path for each anchor point across time. The full model incorporates both the rotated, compressed image data along with a smoothing spline basis:

(1) $${\hat{y}}_{\theta ,b,z}(t)=W_{k,z}\beta_{\theta ,b,z}+\Phi (t)\gamma_{\theta ,b,z}$$

where ${\hat{y}}_{\theta ,b,z}$ indicates the predicted response vector of radii for the anchor point indexed by the given angle, boundary, and horizontal slice respectively. ***W***_*k*_ is represents the pixel image data for image slice z, which has been compressed using the first k principal components. **Φ**(*t*) represents the time-dependent smoothing spline basis, and *β*_*θ,b,z*_ and *γ*_*θ,b,z*_ represent the parameter vectors to be fit for each (*θ*, *b*, *z*) combination. The model was fit using least squares.

#### 2.3.3. Model Variants

We also explored two other model variations for comparison. With model 1 (Equation (1)) given above, the second model (Equation (2)) uses principal components from all z slices combined for each anchor point, instead of only using the horizontal slice of the target anchor point:

(2) $${\hat{y}}_{\theta ,b,z}(t)=W_{k}\beta_{\theta ,b,z}+\Phi (t)\gamma_{\theta ,b,z}$$

This approach was utilized to see if information from the other three slices aids in model prediction. Our third model (Equation (3)) simulates a scenario where the machine learning model is human-assisted:

(3) $${\hat{y}}_{\theta ,b,z}(t)=W_{k}\beta_{\theta ,b,z}+\Phi (t)\gamma_{\theta ,b,z}+\delta \mu_{\theta_{0},b_{0},z_{0}}$$

where *μ*_*θ*_0_,*b*_0_,*z*_0__ is the true radius for the given anchor point, and *δ* is a single additional scalar parameter to be fit. In this case, the model assumes that a user has provided the annotation of a single anchor point, of the 48 total, at the beginning image of each full US dataset. Thus, the model has access to the true target response value for one of the roughly 1500 anchor points to be predicted for a given spatiotemporal location. This approach was taken to see whether a single annotation could significantly help in the prediction of the rest of the anchor points in the video sequence. If effective, this strategy could enhance model accuracy with only minor additional effort from a user.

#### 2.3.4. Measuring Model Performance

To test the effectiveness of the developed models, 100 iterations of Monte Carlo crossvalidations were used on the dataset with a training/validation/test split ratio of 6:1:1. The validation sets were used to select the number of principal components for each permutation of the data, while mean squared error (MSE) and R^2^ values were evaluated on the held-out test set. To provide an additional measure of difference between the models, we performed pairwise *t*-tests across the three models for the MSE within each permutation of the test set for each setting of (*θ*, *b*, *z*). Due to the large test-set sample size (i.e., over approximately 500), assumption of normality of the sample mean difference was considered appropriate. The percentage of *t*-tests with *p* < 0.05 was computed, aggregated across the angles, short-axis slices, and Monte Carlo sample. We note that this does not necessarily provide any statistical guarantees regarding Type I/II error rates, but is intended instead to serve as an additional metric for model comparison.

### 2.4. Description of Metrics Derived from LV Mesh

Once a final 4D mesh of the left-ventricle is created, a series of metrics that characterize its regional kinematics are systematically extracted based on the Lagrangian-definition of linear or engineering strain [28-31] in both the circumferential (Equation (4)) or longitudinal frame (Equation (5)):

(4) $$\varepsilon_{C}(t,z)=\frac{perimeter_{t}-perimeter_{t=0}}{perimeter_{t=0}}$$

(5) $$\varepsilon_{L}(t,\theta )=\frac{length_{t}-length_{t=0}}{length_{t=0}}$$

where each metric is a function of time t (i.e., a given position within the cardiac cycle), and circumferential and longitudinal strain curves are a function of both position z (i.e., location along the *z*-axis) and angle *θ* (i.e., rotation from the positive *x*-axis), respectively. Furthermore, additional metrics can be derived from each curve including the early/late systolic strain rates and early/late diastolic strain rates, providing insight into how the heart is moving between end-diastolic and peak-systolic states.

To assess the robustness and practical use of the machine learning-based predictions of wall kinematics, these metrics were computed at select locations in the circumferential (e.g., basal, mid-LV, and apical) and longitudinal (e.g., anterior, posterior, anterior free-wall and septum, and posterior free-wall and septum) frames. Derived metrics from both the ground-truth and machine learning-predicted boundaries were compared using paired *t*-tests with Bonferroni–Dunn’s multiple comparisons corrections. We note that the distribution of several metrics showed some minor deviations from normality (e.g., slight skewness). Adjusted Shapiro–Wilk tests suggested non-normality for roughly 10% of the cross-validation samples. However, the central limit theorem ensures that even when deviations exist from normality among the individual observations, the sampling distribution of the test statistic converges to a normal distribution with larger sample sizes. The recommended sample size threshold for assuming normality via the central limit theorem is 30 [32]. As the sample sizes used for our *t*-tests were roughly 600, it is safe to assume normality of the computed test statistics.

## 3. Results and Discussion

### 3.1. Model Fitting Results

**Figure 2.**
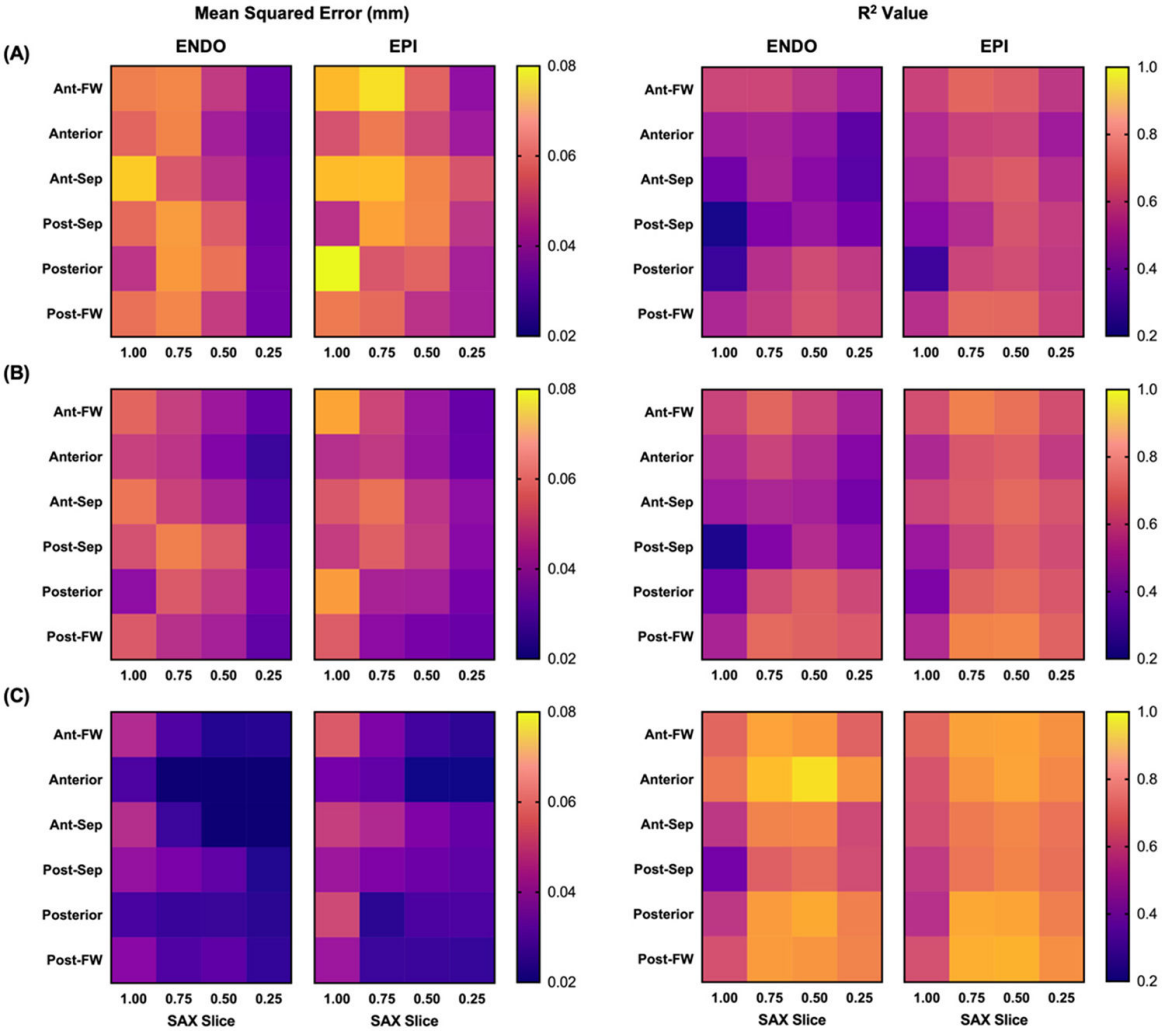
Heatmap representations of median mean-squared-error (left) and associated R values (right) following 100 Monte Carlo simulations of the 6:1:1 testing paradigm, stratified by theta, b, and z. Predictions are based on models (**A**) 1 (i.e., individual z slices), (**B**) 2 (i.e., combined z slices), and (**C**) 3 (i.e., assisted). Specifically here, model 3 was assisted by incorporating t = 0 radius values taken from the endocardial anterior mid-ventricle position (i.e., z = 0.5, theta = 90 deg).

A visual summary of the test set prediction results for models 1, 2, and 3 are demonstrated in Figure 2A-C, respectively. Qualitatively, we can see improvements in R^2^ and mean squared error (MSE) for each successive model. Of note, we observed higher MSE for all three models around the posterior-septum in the basal slice. We believe this may be because the basal septum commonly lies posterior to the sternum and is thus affected by shadowing artifacts. Not only does that make the basal septum harder to annotate, which lowers precision in the ground-truth data, but also the lack of border contrast means it may be incorrectly accounted for in the PCA-based image compression.

Table 1 provides a numerical summary of the three models’ performance. We notice that with regard to every metric in Table 1, model 2 outperforms model 1, and model 3 outperforms model 2. Taken together, these results suggest that simultaneously incorporating all four slices in the prediction model (model 2) yields modest but noticeable improvement over using individual slices (model 1). Furthermore, annotation of a single point at t = 0 (model 3) can significantly improve model predictive accuracy above the previous versions not incorporating user annotations.

**Figure 3.**
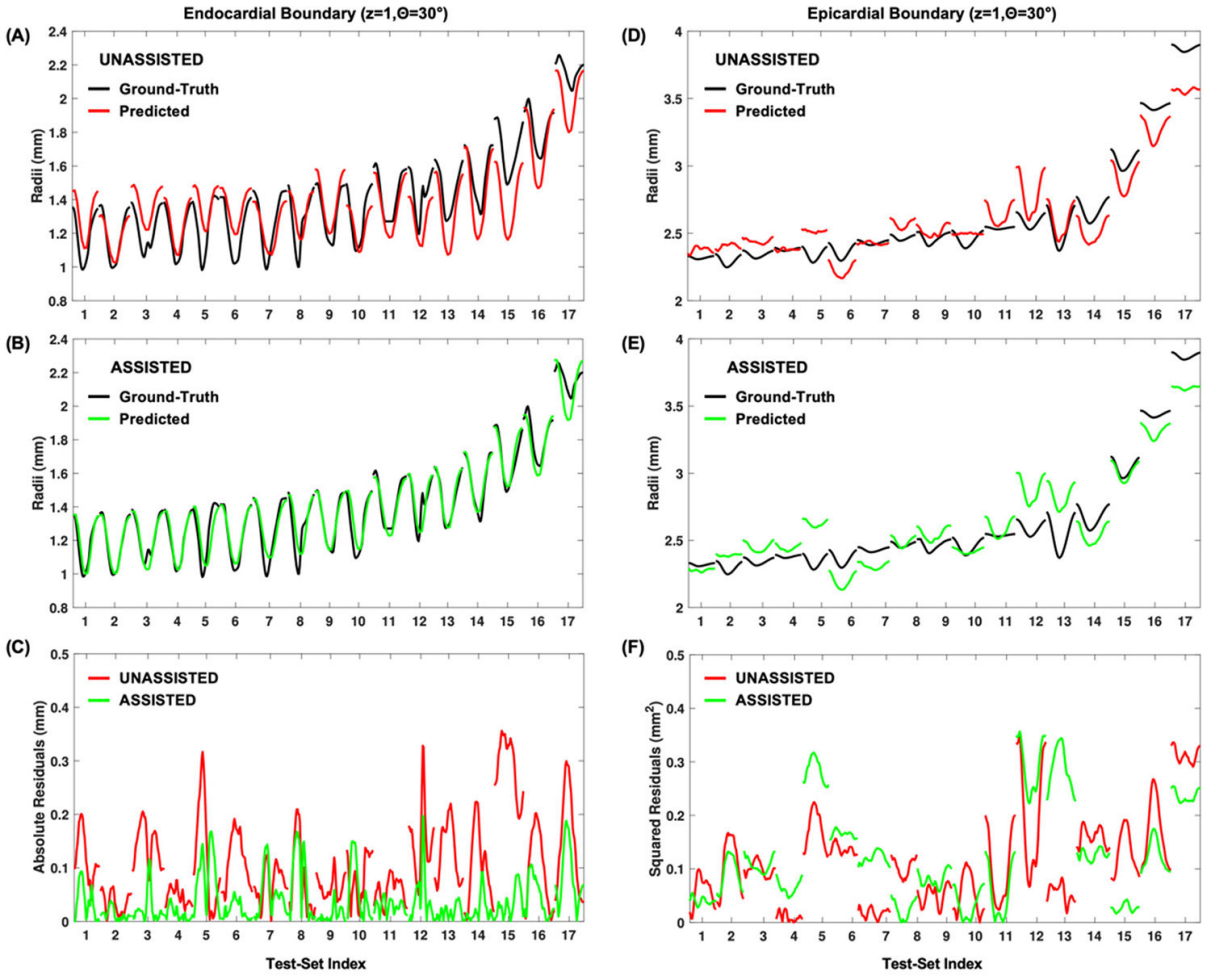
Example predictions on a series of anterior free-wall test set data, based on the implemented 6:1:1 Monte Carlo cross-validation. Raw predictions for 17 separate mice are shown overlaid onto the ground-truth data for unassisted (model 2) predictions at both the (**A**) endocardial and (**D**) epicardial borders, as well as for the (**B**,**E**) assisted (model 3) predictions. Squared error plots at each temporal sample for the (**C**) endocardial and (**F**) epicardial positions demonstrate the potential lower errors resulting from the assisted approach.

While Figure 2 and Table 1 summarize aggregated model performance across all 100 test-set permutations, it also is instructive to see a particular example of the models’ predictions vs. the ground truth. Figure 3A shows the predicted vs. actual radii for the 30° endocardial anchor point at the base of the heart plotted for a single test set using model 2. Qualitatively, model 2′s predictions overall appear relatively close to the ground truth. Errors in predictions seem to be mainly due to offsets in the size of the heart, rather than the wall kinematics (i.e., curve shape is correct but placed off from border). Figure 3B,E shows the accuracy gained using model 3 by incorporating user-assistance on an endocardial point. The heart size is more accurately inferred in this case, yielding better predictions overall. These results illustrate that qualitatively, both the unassisted and assisted models show reasonable performance for both the endocardial and epicardial boundaries on unseen data, with slightly higher accuracy for the endocardial boundary (Figure 3A,B vs. Figure 3D,E). Additionally, although the unassisted model’s predictions are relatively close to the ground truth (Figure 3A,D), the assisted model yields noticeably improved performance, especially for the endocardial boundary (Figure 3B).

### 3.2. Performance of Predication-Based Metrics

Aiming to assess the practicality of using the three proposed models to characterize cardiac function, we computed all metrics described in Section 2.4 and compared results based on predicted and ground-truth boundaries. The results of paired *t*-tests on all 80 metrics, with Bonferroni–Dunn’s multiple comparisons corrections, are shown in Supplemental Table S1, stratified by model. Following trends observed in Section 3.1, the number of metrics that were flagged as significantly different (i.e., adjusted *p* < 0.001) from the ground-truth values were relatively low amongst models 1 (8/80), 2 (7/80), and 3 (8/80). Interestingly, seven metrics showed significant differences regardless of model, suggesting further refinement of the modelling approach would be needed to trust those values if based on machine learning predictions alone. As shown in Table S1, these metrics were all variants of early diastolic strain rate: (1) circumferential early diastolic strain rates at the mid-ventricle and apex; and (2) longitudinal strain at each of the six rotations around the *z*-axis, except at the posterior-septum. While it is not clear why early diastolic strain rate has trouble being properly inferred using our methods, it may be due to the incorporation of severely diseased mice with abnormal diastolic kinematic profiles adversely skewing the imposed temporal-smoothing.

It should be noted that no global function metrics (i.e., end-diastolic or peak-systolic volumes, ejection fraction, or stroke volume) or peak-strain values showed any statistically significant differences between the gold-standard and prediction-based analyses. These results appear promising as the errors are only observed with strain-rate values, specifically early diastolic strain rate, suggesting that the machine learning model predictions of left-ventricle boundaries can be used reliably to assess both global cardiac function and peak circumferential and longitudinal strain. Nevertheless, it is important to note that accurate measurements of early diastolic strain rate are critical to the assessment of diastolic dysfunction [33]. Future work increasing model complexity or implementing novel strategies is thus critical to providing more reliable assessments of cardiac kinematics and function for researchers and clinicians.

### 3.3. Limitations

**Figure 4.**
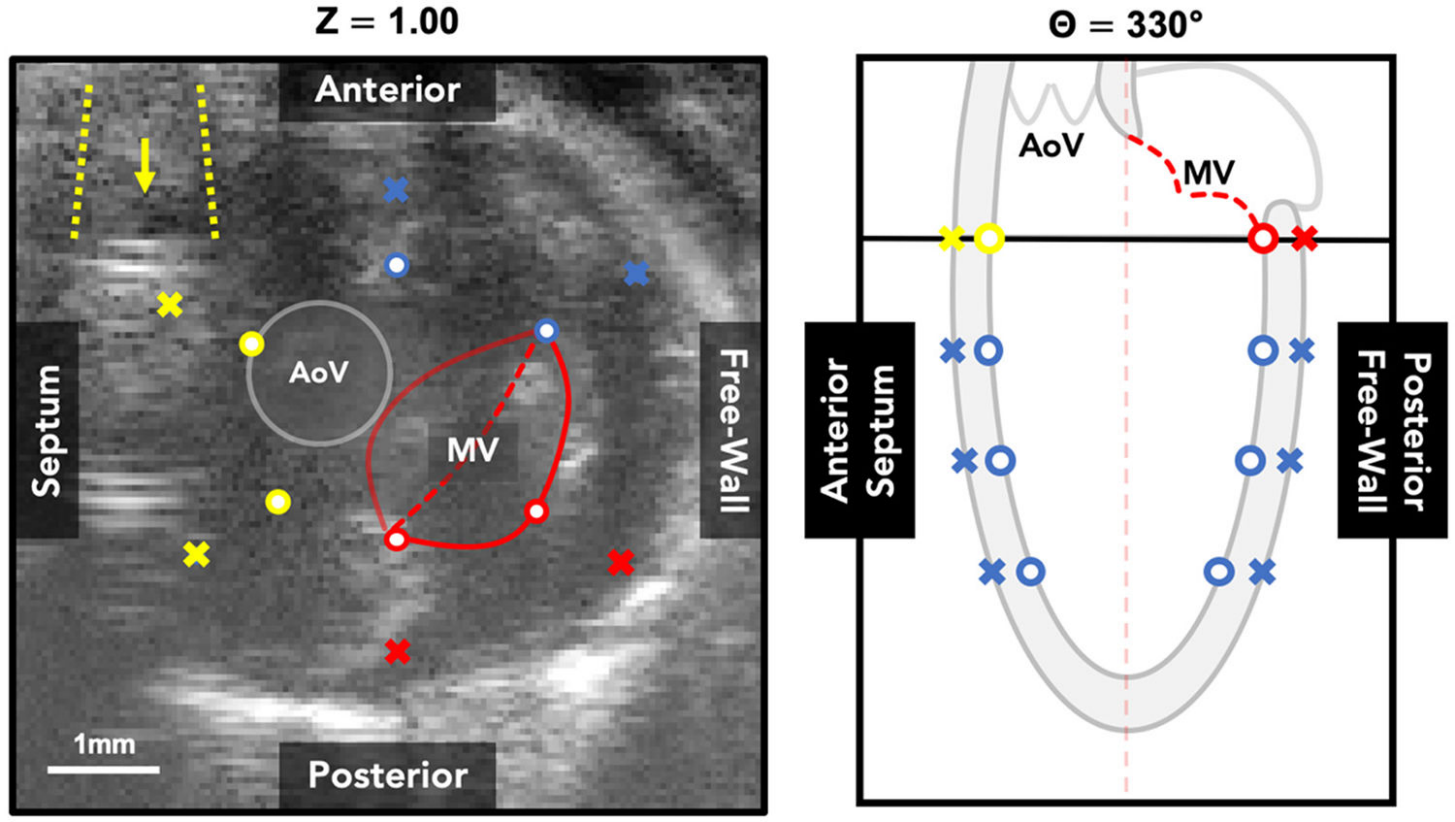
Example short-axis ultrasound image at the base of the heart demonstrating the location of sternum shadow artifacts (yellow) and mitral valve attachments to the left ventricular myocardium (red). Points most commonly affected are color-coded and displayed on a corresponding long-axis schematic.

While we are able to measure model performance, more thorough assessment of interobserver variability in creation of ground-truth could be used to give further confidence in the physiological accuracy of detected boundaries. For example, if model predictions were well within the range of different users’ annotations, then this would give further support to the model’s capability. Furthermore, as seen in Figure 2 and visualized in Figure 4, the septal and posterior walls at the base of the left ventricle are susceptible to prediction errors due to the presence of sternum shadow artifacts and the mitral valve and myocardium interface, respectively. The shadow artifacts can be mitigated by angling the ultrasound probe during acquisition; however, this must be traded-off with undesirable air-based artifacts from the left lung. Additionally, while the mitral valve blending into the myocardium can reduce local contrast, carefully selecting a base location just inferior to the interface can help maintain a proper view of the endo- and epi-cardial borders.

Another natural limitation in working with medical image data is the tendency to have small sample sizes relative to the dimensionality of the data. While we did have a moderate number of videos to work with—136 in total with 30–40 temporal samples—this is relatively few compared to several standard machine learning datasets (e.g., the ImageNet database containing 14 million plus images), but future work will be needed increase the number of training datasets used to build these models. Larger sample sizes not only improve the performance of a given model, but allow for more flexible (i.e., higher dimensional) models to be trained with less risk of overfitting.

### 3.4. Future Applications

As more data are gathered, several avenues exist for extensions or alternatives of the methods proposed in this paper. Notably, with a larger number of images, the use of deep learning for image segmentation, especially convolutional neural networks, would likely become a promising option to explore. Even with moderate sample sizes, the use of deep generative models such as generative adversarial networks (GANs) [34] may provide a mechanism to augment the true dataset with near-realistic images that would enhance model training. GANs have already shown promise in several areas of medical imaging [35-38], and thus would be a natural choice for generation of realistic-looking murine US images. Other data augmentation strategies that have been proposed for medical image generation specifically, such as ASNG [39], could be explored as well.

Transfer learning [40] may be a viable option that could be applied even without additional real or generated data, incorporating a pre-trained network from another application domain. Finally, if a network were developed that could segment murine cardiac images with very high accuracy, it is likely that such a model would be useful in clinical applications, even if downstream transfer learning is only used to fine-tune models on human cardiac images.

Another aspect of this work that could have a more direct impact on clinical translation is the use of a structured grid to sample cardiac kinematics (i.e., four-slices across and six rotations around the LV). While recent studies into 3D speckle-tracking echocardiography have shown promise in characterizing clinical data [41], contours of the left-ventricular boundaries are often unstructured and tracking speckle-patterns is susceptible to error propagation if there is subpar image quality or notable image artifacts. Using an approach similar to the one presented here could allow for the problem of boundary predictions to be simplified and lead to more robust results when speckle-tracking is insufficient, which is a common obstacle associated with murine 4DUS data analysis.

## 4. Conclusions

We demonstrate here the first application of machine learning to the prediction of left-ventricular wall boundaries in murine 4DUS image data. Our results demonstrate notably better agreement between ground-truth and predicted locations when using a model based on a combination of parallel short-axis images compared to treating all images separately. This agreement of predicted locations can then be marginally improved further when incorporating a single boundary point starting location into the model. Furthermore, our results suggest that reliable assessments of global cardiac function and strain, except early diastolic strain-rates, can be derived from the machine learning predictions alone. While future work will aim to strengthen the model efficiency and account for additional murine cardiac disease models, this study reveals that incorporation of machine learning can help vastly increase the reliability and speed of murine cardiac 4DUS data analysis.

## 5. Patents

Patent Pending: “FOUR-DIMENSIONAL IMAGING SYSTEM FOR CARDIOVASCULAR DYNAMICS” (USPTO # 16/903,039).

## Supplementary Material

SuppTable1

## Figures and Tables

**Table 1.** Numerical comparison of model performance for both endocardial and epicardial boundaries. With each model, mean test-set mean squared error (MSE) with associated standard deviations and R^2^ values are displayed. Additionally, the percentage of significant *t*-tests when the given row’s model’s test set MSE is smaller than that of the model indicated in the column is also provided.

	Endocardial				Epicardial			
	MSE (mm^2^)	R^2^	vs. M1 (%)	vs. M2 (%)	MSE (mm^2^)	R^2^	vs. M1 (%)	vs. M2 (%)
Model 1	0.069 ± 0.054	0.41	—	—	0.068 ± 0.044	0.51	—	—
Model 2	0.060 ± 0.049	0.49	48.7	—	0.058 ± 0.039	0.59	54.4	—
Model 3	0.030 ± 0.021	0.71	88.0	83.5	0.037 ± 0.020	0.71	81.9	71.3

## Data Availability

The data presented in this study are available on request from the corresponding author.
